# Relationship Between Empathy, Moral Sensitivity, and Spiritual Care Competence Among Nurses: A Cross-Sectional Study

**DOI:** 10.1155/jonm/8245283

**Published:** 2025-05-18

**Authors:** Yanjia Li, Xin Wen, Yanyun Su, Xiaoying Zeng

**Affiliations:** ^1^Emergency Department, Sichuan Taikang Hospital, Chengdu 610213, Sichuan, China; ^2^Neurology Department, Yibin Second People's Hospital &West China Hospital of Sichuan University Yibin Hospital, Yibin 644000, Sichuan, China; ^3^Gynecology and Obstetrics, Sichuan Taikang Hospital, Chengdu 610213, Sichuan, China; ^4^Neurology Department, Ziyang Central Hospital, Ziyang 641300, Sichuan, China

## Abstract

**Background:** As the global population ages and the number of patients with chronic illnesses increases, caregivers need to consider not only the physical and psychological health of patients but also their spiritual needs when providing care. In providing spiritual care, caregivers must have empathy, moral sensitivity, and spiritual care competence. By exploring the relationship between the three variables, it is essential to improve the overall quality of care and promote patient recovery.

**Aims:** The purpose of this study was to explore the relationship between empathy, moral sensitivity, and spiritual care competence of nurses.

**Methods:** A total of 390 nurses from three hospitals in China were surveyed using an online questionnaire that included nurses' sociodemographic, empathy, moral sensitivity, and spiritual care competence. The data were analyzed using statistical software IBM SPSS 27.0.

**Results:** The results showed that the total score of nurses' spiritual care competence was 47.92 ± 8.71. The relationship between empathy, moral sensitivity, and spiritual care competence was significant, with correlation coefficients ranging from 0.400 to 0.574 (*p* < 0.01). The results of multivariate analysis show that empathy, moral sensitivity, and other variables were the main predictors of nurses' spiritual care competence, explaining 30.3% of the total variation.

**Conclusions:** This study found that empathy and moral sensitivity of nurses may be an effective way to improve nurses' spiritual care competence. The results of this study not only provide reference for improving the spiritual care competence of nurses but also improve the quality of care and patient care experience.

**Implications for Nursing Management:** The results of this study suggest that nursing managers and educators should pay attention to nurses' empathy and moral sensitivity and take timely intervention measures to improve nurses' spiritual care competence. To achieve the goal, comprehensively improving the professional quality of nurses and promoting the development of whole-person nursing is essential.

## 1. Introduction

As the global healthcare landscape continues to transform, the requirements for patient care are growing more varied and intricate. The nursing profession is increasingly recognizing the importance of holistic care, where nurses' roles extend beyond addressing physical health to also consider patients' psychological, social, and spiritual dimensions [1]. In particular, when patients suffer from serious illnesses, their spiritual needs are more urgent [2]. Spiritual care is care that identifies and responds to an individual's spiritual needs in the face of a significant life event or grief, spiritual needs exist at all instances of life, but they may be more sensible in particular situations such as illness, loss and grief [3]. Although introduced to China recently, spiritual care has gained prominence in modern society as awareness of mental health and spiritual well-being has increased [4]. Nowadays, it has gradually become one of the most important concerns in nursing practice. Studies show that spiritual care significantly impacts patients' mental, emotional, and spiritual health [5]. It also influences their perception of illness, quality of life, and treatment outcomes [6]. Given that nurses play a crucial role in patient care, developing the skills to provide effective spiritual care—through meaningful communication, emotional support, and addressing spiritual needs—has emerged as a key competency in contemporary nursing practice, that is, spiritual care competence.

Spiritual care competence refers to an individual's ability to assess and identify a patient's spiritual needs and implement appropriate spiritual interventions to promote the patient's spiritual health [7]. In recent years, there have been more and more research studies on nurses' spiritual care competence. Studies have revealed that both globally and within China, nurses generally exhibit limited spiritual care competence, with Chinese nurses showing particularly lower levels of proficiency [8]. Multiple factors influence nurses' spiritual care competence, including personal attributes (such as education, religious beliefs, and clinical experience), occupational elements (such as workload, professional development, and institutional support), and patient-related aspects (such as cultural background, disease characteristics, and communication openness) [3]. However, the existing research has primarily concentrated on objective factors while giving less consideration to subjective psychological influences. Evidence indicates that psychological factors play a significant role in shaping nurses' spiritual care competence in various ways [9]. These factors determine whether nurses are motivated to allocate emotional resources toward understanding patients' spiritual needs [9]. Moreover, psychological factors influence how nurses respond to patients' challenges. Nurses with positive psychological traits tend to employ more effective care strategies [10]. Therefore, it is of great significance to understand the influence of psychological factors, such as empathy and moral sensitivity on nurses' spiritual care competence to improve nursing quality and promote patients' physical and mental health.

Empathy is generally understood as an individual's ability to recognize and understand the emotions experienced by others [11]. In the field of nursing, empathy requires nurses to be able to put themselves in the patient's shoes, understand their emotions and needs, and take appropriate action in response [12]. Based on Batson's empathy framework, empathy functions not only as an emotional response but also as a driving force for alleviating the suffering of others [13]. The empathetic nature of nurses enables them to accurately assess patients' emotional conditions and deliver effective emotional support, thereby enhancing their spiritual care competence [14]. Furthermore, research has shown that nurses with high empathy are more acutely aware of the spiritual needs of patients, understand the emotional distress that patients may experience during the course of their illness, and by understanding the fear, pain, and helplessness of patients, provide more nuanced and compassionate care [15]. This care is not only physical support but also psychological and spiritual comfort [16]. Therefore, we speculated that the empathy of nurses in this process may have played a role in promoting their spiritual care competence.

Moral sensitivity is an individual's capacity to recognize and discern ethical or moral issues in the context of moral conflicts or dilemmas [17]. For nurses, moral sensitivity encompasses not only adherence to ethical standards but also the ability to make ethically sound decisions in complex nursing scenarios [18]. This capability is crucial for ensuring high-quality care and safeguarding patient rights [19]. According to Leming's Moral Sensitivity Model, moral sensitivity is influenced not only by an individual's ethical framework but also by their emotional intelligence and social interaction skills [20]. Nurses' moral sensitivity can significantly impact their perception and response to patients' spiritual needs [21]. Studies have demonstrated that enhancing nurses' moral sensitivity improves their awareness of patients' spiritual needs [22]. In the course of nursing, it is essential for nurses to focus not only on patients' physical health but also to promptly identify and address their spiritual needs, such as the quest for meaning in life and fear of death [23]. Nurses with higher levels of moral sensitivity are better equipped to accurately assess patients' psychological and emotional states, understand their beliefs and cultural differences, and consequently develop more personalized care plans [20]. This spiritual care, grounded in moral sensitivity, can substantially enhance patients' overall treatment experience. Therefore, we speculate that there may be some connection between the moral sensitivity of nurses and their spiritual care competence.

Although the existing research has examined the importance of empathy, moral sensitivity, and spiritual care competence in nurses, the empirical relationship among these three constructs remains underexplored in the Chinese cultural context. Specifically, integrating empathy and moral sensitivity to enhance nurses' spiritual care competence remains a pressing research question. Of particular concern is the fact that the framework of spiritual care competence, which is rooted in Western religious philosophy, encounters significant cultural tension in the Chinese medical environment, which is grounded in the Confucian ethical system [24]. Traditional Chinese medical ethics take “medicine is the art of benevolence” as the core, emphasizing the hierarchical pattern of medical relations and the responsibility of human relations, which forms a structural difference from the Western spiritual care paradigm dominated by the spiritual needs of patients [24]. Existing studies have shown that Chinese patients' spiritual needs are mostly manifested as realistic questioning of the meaning of life and deep concern for interpersonal harmony, rather than the transcendental religious appeals common in the West [25]. This cultural uniqueness also brings different challenges to improve the spiritual care competence of nurses in China. Most previous studies have ignored the unique cultural background of our country and have not discussed the localization of spiritual care competence.

Therefore, this study aims to investigate the interrelationships between nurses' empathy, moral sensitivity, and their spiritual care competence in the context of Chinese culture. The findings will not only provide theoretical support and practical guidance for nursing managers and educators to improve nurses' spiritual care skills but also assist nurses in better addressing patients' individual spiritual needs, thereby enhancing overall quality of care and quality of life. Additionally, the improvement of spiritual nursing competence has a positive effect on improving nurses' mental health and alleviating job burnout and also contributes to the theoretical innovation of nursing discipline.

## 2. Methods

### 2.1. Design and Setting

A cross-sectional questionnaire design was used in this study. Considering the exploratory nature of the study and time and resource constraints, we used convenience sampling to select 390 nurses from three hospitals in China from April to June 2024. With the consent of the heads of each hospital, we conducted an online questionnaire at each hospital. These hospitals were chosen because they are important partners in our research projects.

### 2.2. Participants

The goal of the study was to recruit registered clinical nurses at three hospitals in China. The inclusion criteria were as follows: nurses (i) aged > 18 years, (ii) who obtain the professional qualification registration of nurses, (iii) who voluntarily participated in this study, and (iv) whose working time is more than six months. The exclusion criteria are as follows: nurses who were not on duty during the (i) survey period and (ii) withdrawal of researchers. The G-power 3.1 program was used to determine the minimum sample size required for the study, with *α* set at 0.05, effect size (*f*^2^) at 0.15, power (1 − *β*) at 0.95, and the number of predictors at 5. It turned out that 139 participants were needed. To account for a possible 20% attrition rate, we collected data from 390 nurses.

### 2.3. Instrumentation Participants

This study used four measures to collect data: the Spiritual Care Competence Scale (SCCS), the Jefferson Scale of Empathy–Health Professionals (JSE-HP), The Moral Sensitivity Questionnaire (MSQ), and a questionnaire for the general characteristics of the participants.

In this study, participants' general characteristics questionnaire was made by reviewing the literature and expert consultation. The questionnaire includes 13 items, such as gender, age, marital status, education level, professional title, and other demographic items.

The SCCS created by van Leeuwen et al. in 2009 [26] was adapted into Chinese by Hu et al. [27]. This version of the scale includes three main dimensions: assessment, implementation, specialization, and quality improvement of spiritual care (12 items); individual and group support (nine items); and patients' spiritual attitude and communication (six items). Participants rate their responses on a 5-point Likert scale, with possible total scores ranging from 27 to 135. In our study, Cronbach's alpha reliability coefficient for this scale was found to be 0.980.

The JSE-HP, developed by Hojat et al. [28] in 2001, is designed to assess the empathy levels of healthcare professionals. An et al. [29] adapted this scale into a Chinese version, which includes 20 items distributed across three dimensions: perspective taking (10 items), compassionate care (seven items), and walking in patient's shoes (two items). Responses are scored using a seven-point Likert scale, ranging from “*strongly disagree*” to “*strongly agree*,” with scores assigned from 1 to 7. The overall score can range from 20 to 140 points, where a higher score reflects greater empathic ability. In our study, Cronbach's alpha reliability coefficient for this scale was found to be 0.789.

The MSQ, originally developed by Lutzen et al. [30], was adapted into Chinese and revised as the Moral Sensitivity Questionnaire–Revised Version for Chinese populations (MSQ-R-CV) by Huang et al. [31]. This questionnaire is utilized to evaluate the moral sensitivity of participants. It comprises two dimensions: moral responsibility and strength (five items) and moral burden (four items), totaling nine items. Each item employs a 6-point Likert scale, ranging from “*strongly disagree*” to “*strongly agree*,” with corresponding scores from 1 to 6. The overall score can range from 9 to 54 points, where higher scores reflect greater moral sensitivity in individuals. In this research, Cronbach's alpha reliability coefficient for the scale was found to be 0.794.

### 2.4. Data Collection

All data were collected through the online questionnaire platform “SoJump.” Prior to the investigation, researchers within each unit underwent training. The researchers strictly screened potential survey subjects based on inclusion and exclusion criteria, followed by sending a survey notice via WeChat, a social software platform widely used in China, informing surveyed nurses about the study's purpose, significance, as well as instructions regarding timing and attention points for completing the questionnaire. Upon receiving respondents' consent, online questionnaires were distributed through provided links to collect data. To ensure data accuracy and validity, participants were unable to submit incomplete questionnaires. In total, 400 questionnaires were sent out during this survey with 390 valid responses collected, resulting in an effective recovery rate of 97.5%.

### 2.5. Data Analysis

Data were analyzed using IBM SPSS 27.0. Normal distributions for nurses' spiritual care competence, empathy, and moral sensitivity were examined using quantile–quantile plotting. Descriptive analysis of all data was performed using percentages, averages, and standard deviations. The relationship between major variables was compared using Pearson correlation analysis. The *t* test and one-way ANOVA analysis were used to compare the demographic differences in nurses' spiritual care competence. Multivariate regression was used to analyze the influencing factors of nurses' spiritual care competence. The statistical significance was set at *p* < 0.05 with two-tailed testing.

### 2.6. Ethical Considerations

This research was approved by the Ethics Committee of Sichuan Taikang Hospital (approval number: SCTK-IRB-2024-030). Participants completed the questionnaire anonymously, and the aims and importance of the study were thoroughly explained to them prior to their participation. Each participant received an online informed consent form prior to joining the study. To ensure the confidentiality of the data, all participants have access to the questionnaire only once, and the data are stored in a password-protected electronic database with access fully restricted to the lead researcher and one authorized researcher.

## 3. Results

### 3.1. Participant Characteristics

The study included 390 participants, of whom 93.1% were female and 63.8% were married. Of them, 67.3% had 6 years or more of work experience, 73.3% had a bachelor's degree or above, and 54.1% held a junior title or below. The remaining information is detailed in Table 1.

### 3.2. Single Factor Analysis of Spiritual Care Competence

The differences in the nurses' spiritual care competence with the number of night shifts/month, overtime work status, spiritual care training, traumatic childhood experience, and their feelings to their family atmosphere were observed (*p* < 0.05) (Table 1).

### 3.3. Descriptive Statistics of Main Study Variables

In this study, the total mean score of nurses' spiritual care competence, empathy, and moral sensitivity were 107.59 ± 18.08, 91.51 ± 18.65, and 40.19 ± 7.18 (Table 2).

### 3.4. Correlation Between Nurses' Spiritual Care Competence, Empathy, and Moral Sensitivity

The results of the Pearson correlation indicated that spiritual care competence was positively correlated with empathy (*r* = 0.400, *p* < 0.01) and moral sensitivity (*r* = 0.453, *p* < 0.01). The empathy was also positively correlated with moral sensitivity (*r* = 0.574, *p* < 0.01) (Table 3).

### 3.5. Multivariate Analysis of Spiritual Care Competence and Its Associated Factors

In this study, nurses' spiritual care competence score was used as the dependent variable, and the number of night shifts/month, overtime work status, spiritual care training, traumatic childhood experience, and their feelings to their family atmosphere, empathy, and moral sensitivity were used as the independent variables for multivariate analysis. The results showed that often work overtime, how is your family atmosphere, whether to attend spiritual care training, empathy, and moral sensitivity were the main predictors of nurses' spiritual care competence, explaining 30.3% of the total variation, this effect has moderate explanatory power (Table 4).

## 4. Discussion

Nurses play a crucial role in providing spiritual care. Spiritual care extends beyond traditional physical and psychological care, emphasizing the fulfillment of patients' spiritual needs during illness, pain, and even at the end of life. This study found that nurses' spiritual care competence was moderate, aligning with the findings of Han et al. [24]. Despite increasing attention to spiritual care in nursing education and clinical practice, many nurses still lack systematic or effective training in this area. Although two-thirds of the nurses in this study had received some form of spiritual care training, their overall spiritual care competence remained relatively low. In China, the severe shortage of nurses and the high-pressure work environment contribute to a focus on physiological treatment and nursing, often at the expense of spiritual care [25]. Additionally, the busy clinical workload limits nurses' opportunities for meaningful interactions with patients, thereby impacting the quality of spiritual care [32]. The current state of nurses' spiritual care competence highlights a significant challenge in contemporary nursing practice. While the importance and value of spiritual care are increasingly recognized, challenges such as inadequate education and training, high work pressure, and skill deficiencies persist [33]. To enhance nurses' spiritual care competence, nursing managers and educators must prioritize spiritual care education and training, improve working conditions, and foster emotional support and teamwork among nurses [34]. These measures will enable nurses to better address patients' spiritual needs and elevate the overall quality of care.

The results of this study confirmed a positive correlation between nurses' empathy and their spiritual care competence. This finding underscores the critical role of empathy in enhancing spiritual care. First of all, this discovery not only holds a significant practical value but also provides a new perspective for the development of nursing and psychology theories. This study verified Batson's empathy framework through empirical data. From the perspective of theoretical integration, this study constructed a three-level action model of “empathy–emotional connection–spiritual care.” Secondly, this study revealed the special manifestation mechanism of emotion contagion theory in the nursing field—nurses achieved comprehensive perception of patients' spiritual needs through the dual-channel effects of cognitive empathy (understanding the patient's perspective) and emotional empathy (sharing emotional experiences). This discovery expanded the application boundaries of social cognitive theory in professional care contexts. Specifically, nurses with a high level of empathy are more likely to understand the emotional needs and psychological states of patients, learn to put themselves in their shoes, experience their pain and confusion from the perspective of patients, understand their unique views on life, health, pain, and death, and empathize with them emotionally. They can implement emotional care for patients [35]. Additionally, by establishing an emotional connection with patients, nurses are able to more accurately identify patients' spiritual needs and provide more targeted and personalized spiritual care [36]. For instance, through active listening, empathetic responses, and support, nurses can effectively alleviate patients' inner distress, offering emotional comfort and support, thereby reducing their emotional burden and promoting spiritual well-being [37]. During the process of providing spiritual care to patients, nurses continuously accumulate spiritual care experience, thereby enhancing their spiritual care competence. Therefore, it is recommended that nursing managers and educators prioritize the development of empathy among nurses, which can be achieved through scenario simulation, communication skills training, psychological support, self-regulation training, group interaction, and reflective learning [14]. These efforts will enhance nurse's emotional resonance and improve their spiritual care competence and patient satisfaction.

The results of this study demonstrate a significant positive correlation between nurses' moral sensitivity and their spiritual care competence. This discovery was systematically explained through the three-stage theoretical framework of Leming's Moral Sensitivity Model. The three-level advanced theory of principle sensitivity, relationship sensitivity, and system sensitivity proposed by Leming's Moral Sensitivity Model provides a mechanistic interpretation path for the results of this study, promoting the theoretical expansion of this model in the field of spiritual care [20]. For example, in the principle sensitivity stage, nurses mainly rely on established ethical norms to make decisions; however, nurses with higher levels of moral sensitivity can promptly identify patients' attitudes and better assess patients' spiritual needs. In addition, in the relationship sensitivity stage, nurses with high moral sensitivity have significantly lower moral perception thresholds, enabling them to detect changes in patients' voice tremor frequency and other extra-linguistic signals, thereby improving their accuracy in recognizing patients' spiritual needs. In the system sensitivity stage, nurses with higher moral sensitivity exhibit more active thinking abilities and continuously improve their cognitive and spiritual care competence. Nurses often face ethical challenges in their clinical practice. For example, when dealing with terminally ill patients, nurses must weigh the expectations of the patient's family against the patient's own wishes and address issues related to prolonging life [38]. Nurses with higher moral sensitivity, who generally have a stronger sense of moral responsibility and awareness, as well as a greater sense of moral burden, is better able to identify these ethical dilemmas keenly and make decisions that are more in the best interests of patients while considering personal needs and ethical principles [39]. In this process, nurses not only provide technical care and understand the spiritual needs of patients but also engage in meaningful emotional exchanges and communication with them, thereby enhancing the effectiveness of spiritual care. Secondly, moral sensitivity allows nurses to recognize cultural differences and the particularities of religious beliefs, ensuring that they do not violate patients' belief systems and strive to deliver culturally appropriate nursing services [21]. Finally, nurses with high moral sensitivity can also identify patients' multilevel needs from the perspective of whole-person care, considering patients' physical, psychological, social, and spiritual well-being in ethical decision-making, thereby providing more comprehensive and personalized care and ultimately enhancing their spiritual care competence [23]. Therefore, it is recommended that nursing managers and educators encourage nurses to reflect on their daily practice through ethical training and education, particularly in situations involving ethical dilemmas and spiritual care, to assist nurses in examining their positions and behaviors in ethical decision-making [40]. Additionally, through the collaboration of multidisciplinary teams, nurses can understand the spiritual needs of patients from a broader perspective and make more informed ethical decisions, ultimately improving the moral sensitivity and spiritual care competence of nurses [41].

The results of this study demonstrated that an increase in nurses' overtime work is associated with a decrease in their spiritual care competence. Given the rising demand in the medical industry and the shortage of nursing staff, nurses frequently have to undertake tasks beyond their regular working hours, making overtime a common occurrence, particularly in China. Prolonged overtime can lead to physical and mental exhaustion, thereby impacting nurses' emotional stability, cognitive function, and the quality of their interactions with patients [42]. Spiritual care, as a critical component of nursing practice, not only requires emotional resonance but also necessitates attention to patients' spiritual needs during working hours [43]. However, research has shown that chronic work stress, excessive overtime, and inadequate rest can deplete nurses' emotional resources, hindering their ability to focus on patients' spiritual needs and thus affecting their spiritual care competence [44]. Therefore, it is recommended that nursing managers allocate working hours more effectively and minimize excessive overtime [45, 46]. Ensuring adequate rest and recovery time will enable nurses to maintain sufficient energy and emotional well-being, thereby enhancing their spiritual care competence and better meeting patients' spiritual needs, ultimately improving the overall quality of care.

Family atmosphere is also one of the critical factors influencing nurses' spiritual care competence in this study. Specifically, a more positive home environment correlates with higher levels of spiritual care competence among nurses. The family serves as the foundation for personal, emotional, and psychological development, subtly shaping nurses' emotional states and professional attitudes [47]. A supportive family atmosphere enhances nurses' emotional resilience and equips them to better manage professional stress. The research indicates that emotional support skills are integral to spiritual care, enabling nurses to build intimate relationships with patients, recognize their emotional needs, provide necessary comfort, and enhance overall spiritual care [45]. Studies also show that nurses from harmonious family environments tend to connect more empathetically with patients, demonstrating higher levels of empathy and delivering more effective spiritual care [48]. Therefore, to improve nurses' spiritual care competence, healthcare managers should consider the family context of nurses and offer family counseling services and organize family support groups to foster a positive family environment [49]. Additionally, hospitals can provide mental health training to help nurses address emotional and psychological challenges both at home and work, thereby reducing stress and enhancing their spiritual care competence.

Finally, the results of this study demonstrate a positive correlation between nurses having received spiritual care training and their spiritual care competence, which aligns with the findings of Yanjia et al. [50]. Nurses who have undergone such training are more adept at identifying patients' emotional and spiritual needs, responding with a warmer and more empathetic attitude, and providing targeted emotional support. This enhances patient satisfaction and trust while strengthening the nurses' spiritual care competence. Moreover, the research indicates that spiritual care training equips nurses with effective stress management and emotional regulation skills, thereby alleviating job burnout and psychological pressure [51]. Consequently, nurses experience greater job satisfaction and a sense of accomplishment, leading to continuous improvement in their spiritual care competence through practical application. Therefore, it is recommended that nursing managers implement diverse training methods, such as classroom instruction, online learning, case discussions, role-playing, and practical operations [25]. Through a variety of training forms, nurses are able to improve their spiritual care competence through simulated practice based on theoretical learning. Furthermore, it is hoped that future longitudinal research will further explore the relationship between spiritual care training and spiritual care competence, thereby proving the reliability and universality of these findings.

### 4.1. Limitations

The aim of this study was to investigate the relationship between empathy, moral sensitivity, and nurses' spiritual care competence. Employing a cross-sectional survey methodology, the study underscores the significance of practical skills in nursing, with a special focus on enhancing spiritual care competence. The results provide valuable insights for nursing educators and administrators to design targeted interventions that can elevate the overall quality of nursing services. However, several limitations should be noted. Firstly, the cross-sectional nature of the study restricts its ability to establish causal relationships between variables, as it can only identify correlations. Future research directions could extend to longitudinal research methods (which can provide a deeper understanding of the dynamic changes among these factors over time), intervention studies (e.g., how targeted empathy training affects spiritual care competence over time), or qualitative methods (such as nurse interviews, which can provide a deeper understanding of nurses' personal experiences in mental care). Secondly, the sample was confined to hospitals in China, which may limit the generalizability and representativeness of the findings. Spiritual care perspectives and values differ across cultures and countries, potentially affecting assessments of empathy and moral sensitivity. Therefore, future studies should aim for cross-cultural validation and include larger, more diverse samples to corroborate these results. Thirdly, the study relies on self-reported measures, which may introduce social desirability bias. Future studies could incorporate qualitative interviews or direct observation to validate responses. Lastly, the cross-sectional design does not sufficiently account for other influential factors, which could confound the outcomes. Future studies could explore additional predictors, such as workplace culture or personal spirituality.

## 5. Conclusions

This study explored the interrelationship between empathy, moral sensitivity, and spiritual care competence in nurses. The results revealed a substantial positive association among these three elements. By fostering nurses' empathy and moral sensitivity, their spiritual care competence can be effectively enhanced. This study provides a theoretical framework for nursing managers and educators to design and implement targeted strategies and related policies or systems aimed at improving nurses' spiritual care competence and also provide ideas for future related research. It is recommended that nursing leaders and educators focus on cultivating nurses' spiritual care competence. Actively change relevant spiritual care training policies or systems by adopting a structured educational approach to help nurses better understand the needs of patients in clinical practice and provide ethical and humane spiritual care. This not only enhances nurses' spiritual care competence but also improves the quality of care and the patient care experience.

## 6. Implications for Nursing Management

This study reveals a significant positive correlation between empathy, moral sensitivity, and spiritual care competence among nurses, which holds important clinical significance and practical value. This study also found that factors such as frequent overtime work, family atmosphere, and participation in spiritual care–related training can influence nurses' spiritual care competence. Therefore, it is suggested that nursing administrators and educators comprehensively enhance the spiritual care competence of nurses through the following measures: Firstly, design emotional care–related training programs to help nurses improve their communication skills with patients and their ability to understand patients' emotional needs. Simultaneously, incorporate emotional intelligence and empathy training into the nursing education curriculum so that nurses can demonstrate higher empathy in various clinical situations, thereby establishing trusting relationships, better understanding patients' emotional needs, and enhancing nurses' spiritual care competence. Secondly, the cultivation of moral sensitivity should not be overlooked. Nursing managers and educators should establish ethical decision-making training modules or case-based teaching in clinical practice to help nurses become familiar with identifying and handling ethical issues, improving their ethical judgment and decision-making abilities, which in turn can enhance nurses' spiritual care competence. Thirdly, reasonable scheduling to reduce nurses' overtime and attention to nurses' mental health is crucial. Most importantly, it is recommended that hospital administrators can implement systematic changes to standardize spiritual care training at the policy or institutional level, such as establishing certification standards for nurses' spiritual care competency, training specialized nurses in spiritual care, and requiring spiritual care training as an annual compulsory credit for nurses. The content of spiritual care training should encompass foundational theoretical knowledge, context-specific intensive spiritual care training, and the application of localized spiritual assessment tools and others. This approach can not only enhance the quality of nursing services and improve nurse–patient relationships but also further promote the overall nursing experience and physical and mental health of patients.

## Figures and Tables

**Table 1:** Personal characteristics and single factor analysis of spiritual care competence (*n* = 390).

Characteristic	*N* (%)	*M* ± SD	*t/F*	*p*
Gender			0.889	0.382
Female	363 (93.1)	107.88 ± 17.55		
Male	27 (6.9)	103.67 ± 24.19		
Age (years)			0.632	0.676
18–25	69 (17.7)	107.35 ± 12.92		
26–30	107 (27.4)	107.53 ± 20.26		
31–35	73 (18.7)	105.16 ± 18.76		
36–40	81 (20.8)	108.04 ± 20.45		
41–45	39 (10)	109.23 ± 14.68		
46–60	21 (5.4)	112.38 ± 14.64		
Marital status			0.028	0.972
Married	249 (63.8)	107.76 ± 18.89		
Single	131 (33.6)	107.32 ± 16.76		
Divorce or others	10 (2.6)	107.10 ± 15.23		
Education level			1.029	0.304
College	104 (26.7)	109.15 ± 15.60		
University or above	286 (73.3)	107.02 ± 18.89		
Professional title			1.392	0.245
Nurse	89 (22.8)	108.47 ± 14.67		
The primary nurse practitioner	122 (31.3)	107.71 ± 18.85		
Nurse-in-charge	153 (39.2)	105.99 ± 19.55		
Deputy director nurse or above	26 (6.7)	113.46 ± 15.16		
Department			0.596	0.733
Internal medicine	113 (29)	107.69 ± 15.23		
Surgical	33 (8.5)	105.85 ± 19.96		
Pediatric	30 (7.7)	104.87 ± 18.84		
Obstetrics and gynecology	21 (5.4)	112.29 ± 19.50		
Outpatient	40 (10.3)	110.35 ± 20.17		
Emergency	39 (10)	107.97 ± 21.03		
Others	114 (29.2)	106.75 ± 18.01		
Working experience (years)			1.902	0.109
< 3	70 (17.9)	105.80 ± 13.55		
3–5	58 (14.9)	109.50 ± 19.05		
6–10	79 (20.3)	108.16 ± 19.47		
11–15	86 (22.1)	103.92 ± 21.02		
> 15	97 (24.9)	110.54 ± 15.88		
Income (RMB/month)			1.315	0.269
< 3000	26 (6.7)	108.62 ± 12.85		
3000–5000	106 (27.2)	109.61 ± 18.52		
5001–10000	228 (58.5)	106.11 ± 18.51		
> 10,000	30 (7.7)	110.87 ± 16.51		
The number of night shifts/month			2.651	0.049
0	146 (37.4)	108.32 ± 17.72		
1∼4	60 (15.4)	106.03 ± 16.84		
5∼8	64 (16.4)	102.64 ± 17.56		
> 8	120 (30.8)	110.13 ± 18.98		
Overtime work status			3.414	< 0.001
No	243 (62.3)	110.29 ± 18.12		
Yes	147 (37.7)	104.10 ± 17.48		
Spiritual care training			−3.316	0.001
No	71 (18.2)	101.24 ± 16.83		
Yes	319 (81.8)	109.01 ± 18.07		
Traumatic childhood experience			2.552	0.012
No	337 (86.4)	108.5 ± 16.97		
Yes	53 (13.6)	101.81 ± 23.35		
Their feelings to their family atmosphere			9.635	< 0.001
Like	272 (69.7)	110.10 ± 18.30		
Average	109 (27.9)	102.35 ± 15.90		
Dislike	9 (2.3)	95.44 ± 19.59		

**Table 2:** Descriptive analyses of spiritual care competence, empathy, and moral sensitivity (*n* = 390).

Variables	Min	Max	*M* ± SD
*Spiritual care competence*			
Assessment, implementation, specialization, and quality improvement of spiritual care	12	60	47.92 ± 8.71
Individual and group support	9	45	34.55 ± 7.02
The spiritual attitude and communication of patients	6	30	25.13 ± 3.86
Total score	27	135	107.59 ± 18.08

*Empathy*			
Perspective taking	10	70	58.47 ± 8.43
Compassionate care	8	56	26.86 ± 13.37
Walking in patient's shoes	2	14	6.19 ± 3.60
Total score	20	140	91.51 ± 18.65

*Moral sensitivity*			
Moral responsibility and strength	5	30	22.82 ± 4.13
Moral burden	4	24	17.37 ± 7.18
Total score	9	54	40.19 ± 7.18

**Table 3:** Correlation coefficients of the study variables (*n* = 390).

Variables	S	S1	S2	S3	E	E1	E2	E3	M	M1	M2
S	1										
S1	0.957^∗∗^	1									
S2	0.939^∗∗^	0.840^∗∗^	1								
S3	0.817^∗∗^	0.699^∗∗^	0.686^∗∗^	1							
E	0.400^∗∗^	0.360^∗∗^	0.440^∗∗^	0.263^∗∗^	1						
E1	0.651^∗∗^	0.619^∗∗^	0.564^∗∗^	0.626^∗∗^	0.460^∗∗^	1					
E2	0.242^∗∗^	0.210^∗∗^	0.232^∗∗^	0.268^∗∗^	0.895^∗∗^	0.228^∗∗^	1				
E3	0.222^∗∗^	0.205^∗∗^	0.200^∗∗^	0.291^∗∗^	0.778^∗∗^	0.266^∗∗^	0.858^∗∗^	1			
M	0.453^∗∗^	0.428^∗∗^	0.426^∗∗^	0.380^∗∗^	0.574^∗∗^	0.501^∗∗^	0.397^∗∗^	0.327^∗∗^	1		
M1	0.517^∗∗^	0.486^∗∗^	0.481^∗∗^	0.455^∗∗^	0.512^∗∗^	0.549^∗∗^	0.304^∗∗^	0.234^∗∗^	0.920^∗∗^	1	
M2	0.298^∗∗^	0.286^∗∗^	0.287^∗∗^	0.228^∗∗^	0.536^∗∗^	0.354^∗∗^	0.425^∗∗^	0.369^∗∗^	0.903^∗∗^	0.663^∗∗^	1

*Note:* S: spiritual care competence; S1: assessment, implementation, specialization, and quality improvement of spiritual care; S2: individual and group support; S3: patients' spiritual attitude and communication; E: empathy; E1: perspective taking; E2: compassionate care; E3: walking in patient's shoes; M: moral sensitivity; M1: moral responsibility and strength; M2: moral burden.

^∗∗^ *p* < 0.01.

**Table 4:** The multivariate analysis of spiritual care competence and its associated factors (*n* = 390).

Variables	*B*	SE	Beta	*t*	*p*
Constant	61.764	5.501	—	11.227	< 0.001
Empathy	0.210	0.050	0.216	4.183	< 0.001
Moral sensitivity	0.814	0.132	0.323	6.186	< 0.001
Overtime work status	−7.202	1.570	−0.198	−4.588	< 0.001
Their feelings to their family atmosphere	−4.823	1.505	−0.138	−3.205	0.001
Spiritual care training	4.246	2.015	0.091	2.107	0.036

*Note:R* ^2^ = 0.312, adjusted *R*^2^ = 0.303, *F* = 34.773, and *p* < 0.001.

## Data Availability

The data that support the findings of this study are available from the corresponding author upon reasonable request.
